# Gaps in protection: the actual challenge in malaria elimination

**DOI:** 10.1186/s12936-023-04473-x

**Published:** 2023-02-07

**Authors:** Krijn P. Paaijmans, Neil F. Lobo

**Affiliations:** 1 Center for Evolution and Medicine, School of Life Sciences, Arizona State University, Tempe, AZ USA; 2 The Biodesign Center for Immunotherapy, Vaccines and Virotherapy, Arizona State University, Tempe, AZ USA; 3 Simon A. Levin Mathematical, Computational and Modeling Sciences Center, Arizona State University, Tempe, AZ USA; 4 Centro de Investigação Em Saúde de Manhiça, Fundação Manhiça, Maputo, Mozambique; 5 ISGlobal, Barcelona, Spain; 6 Eck Institute for Global Health, University of Notre Dame, Notre Dame, IN USA; 7 Malaria Elimination Initiative, University of California San Francisco, San Francisco, CA USA

## Abstract

Progress in reducing both malaria cases and deaths has stalled with regression seen in many geographies. While significant attention is given to the contributing challenges of drug and insecticide resistance, ‘residual’ malaria is often diminished to transmission resulting from outdoor-biting or zoophagic/opportunistic mosquito vectors. These specific vector bionomic traits are only part of the problem, as residual transmission may be driven by (a combination of) (1) sub-optimal intervention coverage, quality, acceptance, and/or usage, (2) drug resistance, (3) insecticide resistance, (4) refractory, resistant and adaptive vector and human behaviours that lower intervention effectiveness, (5) lack of, limited access to, and/or willingness to use healthcare systems, (6) diagnostic sensitivity along with the parallel issue of *hrp2/3* mutations, (7) (inter)national policy, (8) the research and development pipeline, and (9) external factors such as natural disasters and conflict zones. Towards combating the minimization of this extensive and multipronged issue among the scientific community, funding agencies, and public health officials responsible for guiding or developing malaria programmes, an alternative way of describing this transmission is proposed by focusing in on the causative *‘gaps in protection’*. Defining and wording it as such zeros in on the drivers that result in the observed remaining (or increasing) transmission, allowing the malaria community to focus on solutions by identifying the actual causes. Outlining, defining and quantifying the gaps in protection for a given system is of utmost importance to understand what needs to be done, differentiating what can be done versus what cannot be tackled at that moment, along with delineating the technical and financial capacity required.

## Background

The scaling up of core malaria interventions to prevent onward transmission has led to large reductions in the global malaria burden since 2000. Diagnosis and treatment of clinical malaria cases with artemisinin-based combination therapy, insecticide-treated nets (ITNs)—later replaced by long-lasting insecticidal nets (LLINs)—and indoor residual spraying (IRS) are the three core interventions that are estimated to have averted up to 663 million clinical cases between 2000 and 2015, halved *Plasmodium falciparum* infection prevalence in endemic Africa and reduced clinical disease by 40% [1]. However, malaria cases are on the rise again and progress has stalled [2]. In addition, while the use of recommended malaria control interventions has led to large reductions in the malaria burden in several African malaria elimination settings [3, 4], local elimination has not been achieved.

The plateauing of intervention efficacies resulted in the concept of ‘residual transmission’ gaining significant interest and attention over recent years [5–7]. The World Health Organization (WHO) defined residual malaria transmission in 2016 as ‘persistence of transmission after good coverage has been achieved with high-quality vector control interventions to which local vectors are fully susceptible’. In 2018, this definition was updated to ‘persistence of malaria transmission following the implementation in time and space of a widely effective malaria programme’ [8], indicating a constructive shift in focus away from intervention strategies that only target the mosquito vector. However, the word ‘residual transmission’ denotes that this is ‘remaining (transmission) after the greater part or quantity has gone’, which is not necessarily the case. A large portion of transmission may still be present even after the implementation of recommended interventions—evident in the recent increases of transmission seen [2].

This ‘residual’ malaria is often coupled with transmission resulting from outdoor-biting vectors, or zoophagic/opportunistic mosquitoes (i.e. those mosquitoes feeding primarily on animals) [6, 7]—thereby minimizing and relegating this to being a ‘vector issue’. This is also not necessarily true or a primary causal factor as demonstrated by human behaviour analysis and the association of primary vectors with indoor transmission in areas with high LLIN coverage [9–12]. A primary, but often ignored paradigm, is that the transmission system intrinsically changes when an intervention strategy is implemented. Present recommended monitoring and evaluation frameworks do not generally adapt to this simple concept and the failure to monitor and understand these shifting drivers of transmission results in the inability to appropriately adapt the intervention strategy towards being continuously effective.

**Fig. 1:**
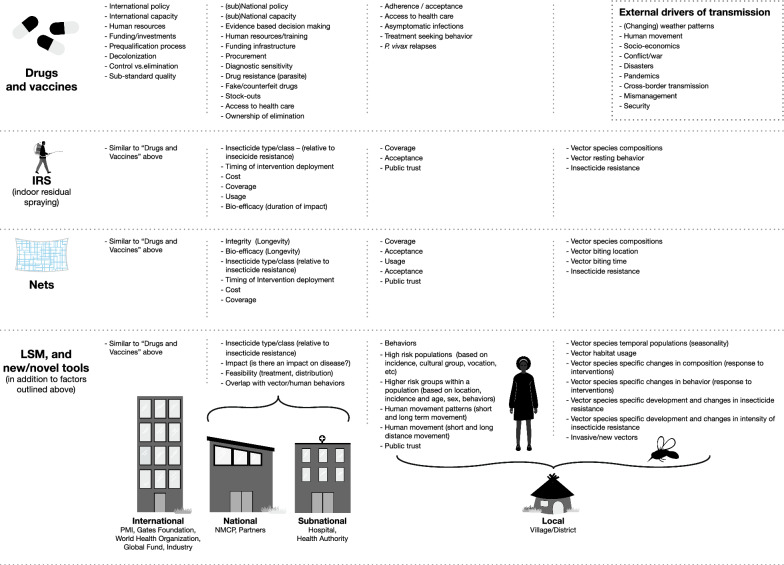
Potential gaps in protection that can hamper malaria control and elimination efforts, across partners, interventions and geographies

Ironically, mosquito and human behaviours may only be part of the problem, as residual transmission may be driven by (a combination of) (1) sub-optimal intervention access, coverage, quality, acceptance, and/or usage [13, 14], (2) drug resistance [15, 16], (3) insecticide resistance [17], (4) refractory, resistant and adaptive vector and human behaviours that lower intervention effectiveness [18], (5) lack of, limited access to, and/or willingness to use healthcare systems [19], (6) diagnostic sensitivity along with the parallel issue of *hrp2/3* mutations [20], (7) (inter)national policy [21], (8) the research and development pipeline [22], and (9) external factors such as natural disasters and conflict zones [23, 24] (Fig. 1). Many of these observed drivers of the transmission presently represent barriers that are harder to combat than just ‘providing one LLIN per two household members’, since they not just represent requirements in technical and infrastructural capacity, but will also require perceptual, cultural, and behavioural shifts in how industry, policy makers, implementers and end-users approach disease elimination.

To decrease this minimization and possible misunderstanding among the scientific community, funding agencies, and public health officials responsible for guiding or developing malaria programs, an alternative way of describing this transmission is proposed here by focusing on the causative *‘gaps in protection’*. This term is used to describe a circumstance when (1) an individual’s malaria infection is not cleared, and (2) an individual and/or household is potentially exposed to malaria infection (i.e. an infective mosquito bite) due to a lack of an effective and/or adequate protective or preventive intervention in place. These two points address both bi-directional (between humans and mosquitoes) malaria transmission and reservoirs, as well as the intervention frameworks that impact each. Most often, gaps in protection can be directly identified through an assessment of how interventions interact with local human and/or vector populations.

For the current interventions targeting the parasite reservoirs (drugs and vaccines), examples of gaps in protection may include undiagnosed cases due to lack of expertise or failing diagnostics, antimalarial stockouts, counterfeit drugs, non-adherence to the treatment regime, and the exclusion of certain populations (e.g., pregnant women, babies or elderly). For the current core vector control intervention (LLINs), examples of gaps in protection include insecticide resistance (reducing the effectiveness of the protection that the insecticide in LLINs provides), occasions when people are outdoors without protection against potentially infective mosquito bites, suboptimal coverage, and usage. For IRS, gaps in protection may include insecticide resistance, vector species that do not rest on sprayed surfaces, duration of IRS impact, spray quality, people washing walls after spraying, and so on.

Other external drivers of transmission that can also contribute to gaps in protection include natural disasters, such as hurricanes and cyclones, or extreme wet years (e.g., due to El Niño/ La Niña) that affect mosquito abundance and species diversity and typical transmission patterns. Conflict zones and the Covid-19 pandemic also result in systemic drivers of gaps in protection impacting both vector intervention frameworks as well as health system functionality.

Focusing on the gaps in protection allows for zeroing in on the drivers that result in the observed remaining (or increasing) transmission, allowing the malaria community to focus on solutions by identifying the actual causes. Outlining, defining and quantifying all gaps for a given system is of utmost importance as it will highlight gaps that can be closed now and gaps for which there is currently no solution, and helps to outline the technical and financial capacity required. Solutions may be as simple as increasing intervention coverage or usage, or the implementation of a supplemental outdoor intervention that combats an outlined gap in protection -a targeted and tailored strategy that addresses local drivers. More systemic and forward-thinking solutions may include a cultural shift within a Ministry of Health or local at-risk communities, and developing novel supply chain mechanisms. It is imperative to outline gaps in protection that simply cannot be closed (i.e. coverage or adherence are at their maximum achievable levels, or there is no present recommended intervention that is suitable for a particular transmission space and time). Understanding the gaps in protection has an additional advantage—enabling appropriate expectations of effect with the implementation of, or change in an intervention strategy. Looking through this ‘gaps in protection’ lens, the implementor can now develop a focused and catered strategy, stratify responses based on characterized drivers and capacity, while also moderating expectations of impact. Consequently, control and elimination strategies may be optimized by adaptively tackling remaining gaps in protection while expectations are adjusted in parallel.

Outlining a strategic plan with all incident data, as well as the gaps in protection, allows for a realistic approach towards malaria elimination, encompassing what has been done, what should be most appropriate to the site, what is actually feasible, and most importantly, what cannot be done at present. This points to strategies, capacity, funding, the need to improve present or develop novel interventions, which are required towards reaching our goal of eliminating malaria.

## Data Availability

Not applicable.
